# The Impact of Technology Teaching in the Dental Predoctoral Curriculum on Students’ Perception of Digital Dentistry

**DOI:** 10.3390/dj12030075

**Published:** 2024-03-13

**Authors:** Leonardo M. Nassani, Sompop Bencharit, Fernanda Schumacher, Wei-En Lu, Rodrigo Resende, Gustavo Vicentis Oliveira Fernandes

**Affiliations:** 1 Division of Restorative and Prosthetic Dentistry, The Ohio State University College of Dentistry, 3005F Postle Hall, 305 W 12th Ave, Columbus, OH 43210, USA; 2 Department of Oral Rehabilitation, Medical University of South Carolina College of Dental Medicine, BSB 335C, 175 Ashley Ave, Charleston, SC 29425, USA; 3 Division of Biostatistics, The Ohio State University College of Public Health, 280A Cunz Hall, 1841 Neil Ave, Columbus, OH 43210, USA; 4 Department of Biostatistics, The Ohio State University College of Arts and Sciences, 305E Cockins Hall, 1958 Neil Ave, Columbus, OH 43210, USA; 5 Faculty of Dentistry, Fluminense Federal University, Niteroi 24020-140, RJ, Brazil; 6 Missouri School of Dentistry & Oral Health, A. T. Still University, St. Louis, MO 63104, USA

**Keywords:** digital dentistry, CAD/CAM, technology, dental students, practice management

## Abstract

The goal was to assess dental students’ perception of digital technologies after participating in a CAD/CAM exercise for scanning, designing, and manufacturing computer-aided provisional fixed dental restorations. A survey was conducted among second- (pre-D2 and post-D2), first- (D1, negative control), third-, and fourth-year dental students (D3 and D4, positive controls). Only OSU College of Dentistry students who completed the activity and completed the surveys were included. Seven questions were rated, which evaluated changes in knowledge, skill, interest, the importance of technology availability in an office, patients’ perception of technology, the importance of having the technology, and the expected frequency of clinics utilizing the technology. Statistical analysis was performed with a significance level of 0.05. A total of 74 pre-D2 and 77 post-D2 questionnaires were completed. Additionally, 63 D1, 43 D3, and 39 D4 participants responded to the survey. Significant differences were found for “knowledge” and “skill” between the pre-D2 and post-D2 and pre-D2 and control groups (*p* < 0.001). There was a significant difference between the post-D2 participants and all the controls in terms of “interest” (*p* = 0.0127) and preference for in-practice technology availability (*p* < 0.05). There were significant results between the post-D2 participants and all the controls regarding the importance of technology availability in an office (*p* < 0.001) and the expected frequency of clinics utilizing the technology (*p* = 0.01). No significance was found for “value of technology to patients” and “the importance of having the technology”. The presence of technology in practice and in educational academic environments significantly improved students’ interest and perception of their knowledge and skill.

## 1. Introduction

The emergence of digital dentistry and dental computer-aided design/computer-aided manufacturing (CAD/CAM) technologies based on oral/dental scans has prompted dental schools to revise their curriculum and integrate dental technologies into the education of dental students [1,2]. CAD/CAM composite restorative materials are now accessible for subtractive fabrication procedures, utilizing milling machines and uniformly manufactured blocks. These blocks exhibit enhanced properties compared to direct composite materials, consisting of a resin polymer matrix and ceramic-based filler particles [3]. The composition, proportions, properties, and distribution of the compartments vary greatly among different CAD/CAM materials, resulting in diverse material characteristics [3,4,5,6,7,8].

Digital impression acquisition (scanning) and CAD/CAM technology, including machine milling and three-dimensional (3D) printing, have significantly advanced modern restorative and prosthetic procedures. Currently, it is possible to print monolithic zirconia as a good option for restoring single crowns [9] and preparing orthodontic appliances [10] and surgical guides [11]. These technological advancements have gained widespread acceptance and are extensively utilized in private dental offices, resulting in positive feedback and transforming clinicians’ perceptions and practice models [4,12,13,14]. However, limited clinical studies specifically investigating composite-based CAD/CAM materials are available. Most of the existing studies are in vitro and primarily focus on mechanical tests, such as fatigue and fracture behavior, predominantly in relation to veneer restorations [15,16,17,18,19,20].

Typically, the resin composites for 3D printing are acrylic-based photopolymers [21]. The addition of ceramic particles and nanoparticles originated a new class named nanohybrid resin composites, allowing the manufacture of permanent dental restorations [22,23]. Other types are self-curing acrylic resin/bis-acryl resin [24]. Adding inorganic fillers modifies the material’s purpose, increasing its flexural strength, elastic modulus, and hardness [25]. Another important characteristic is the thickness of the printed layer; most manufacturers suggest a layer thickness of 50 μm for resin composites to reduce the staircase effect and surface roughness [26]; however, increasing the layer to 100 μm can reduce the printing time by around 40% [27] but may impact the physical and mechanical properties of the printed materials [28].

Incorporating digital technologies into dental education is necessary to keep pace with advancements in materials and technology. However, there is limited literature on how students perceive this integration and how it affects their professional aspirations in private practice. One of the ways to assess how students received and processed the information transmitted is by employing a survey-based evaluation. Thus, a study [29] evaluated the quality of survey-based research reports published in dentistry journals over four years. The authors included 99 articles. The best-reported items were the description of the introduction, the results concerning the objectives, and the presence of an ethics committee; otherwise, some items were poorly reported: declaring the incentives to the study participants, statistical analysis, and information on how the nonrespondents differed from the respondents. The conclusion presented considered the reporting on all aspects that should be considered in survey-based studies in dentistry journals to be of moderate quality; therefore, the poorly reported criteria were found mainly in the statistical analysis. In order to better standardize this type of study, Magnuson et al. [30] suggested guidelines and reminders, including the following points: 1. type of sample of respondents; 2. how the survey was designed; 3. how it was piloted; 4. whom the survey was sent to and how their contact information was obtained; 5. how the survey was distributed and the number and nature of follow-up periods; 6. the response rate as a percentage; 7. the number of completed surveys used for the data; 8. how the data were managed and manipulated, including the software used and methods employed; 9. the inclusion of tables and figures.

Observing all the prerequisites for survey-based studies, the goal of this article was to assess dental students’ perceptions of digital technologies by exposing them to a pre-clinical simulation exercise of scanning, designing, and manufacturing a computer-aided provisional fixed dental restoration. It was proposed here that comprehensive digital dentistry teaching and exposure will influence students to incorporate dental technologies in their future clinical practice. The null hypothesis is that there will be no difference in student perception after exposure to this learning experience.

## 2. Materials and Methods

This study was submitted and approved by the University’s ethical committee (#20220799).

### 2.1. Sample Selection and Grouping

Second-year students typically receive pre-clinical education in Fixed Prosthodontics and Operative Dentistry as part of the dental curriculum at the College of Dentistry. Previously, these students were introduced to digital impression acquisition (intraoral scanning) on typodonts and were exposed to computer-aided design (CAD) without computer-aided manufacturing (CAM) during this pre-clinical training. However, as part of the curriculum re-engineering process, a comprehensive CAD/CAM exercise was developed and implemented in the fourth course of the operative curriculum during the fall semester of the second year. A survey was conducted among the intervention group to evaluate any changes in perception regarding digital dentistry.

### 2.2. Eligibility Criteria

Table 1 shows the inclusion criteria, which consisted of second-year dental students, both before and after completing the comprehensive CAD/CAM exercise. The same survey was also administered to the first-year dental students (who had not yet been exposed to digital dentistry) and the third and fourth-year dental students (who had limited exposure to intraoral scanning and CAD exercises during their pre-clinical training) as control groups. All students from the first, second, third, and fourth years were recruited, and participants who did not complete all the required surveys for each respective group were excluded from the analysis.

All the included students agreed to participate before starting the survey. In terms of inclusion, only students from the OSU College of Dentistry (OH, USA) who accepted being part of the study, completed the activity, and filled out the surveys (initially and after the activity) were considered; students in their first year, who did not learn about Digital Dentistry, filled the survey out as a negative control; and as a positive control, students in their third and fourth years participate. Participants who did not complete the activity proposed at the correct time or did not fill out the survey were excluded.

### 2.3. Sample Size Calculation

For the validation process, the professional dentists (five authors) answered the same survey at two different moments, one week apart. The test–retest reliability was calculated using intra-class correlations (ICCs), where we considered a minimum acceptable reliability (ρ0) of 0.7 and a hoped reliability (ρ1) of 0.9. Using this parameter, the calculated sample size was 65 participants. Considering a drop-out rate of 10%, a minimum of 72 participants was needed.

### 2.4. Activity Proposed

The exercise conducted in the pre-clinical simulation laboratory involved a clinical scenario on a typodont, specifically preparing an ivorine molar tooth #46 (Nissin Dental Products—Kilgore, Kyoto, Japan) for CAD/CAM onlay restoration. The process began by acquiring a digital impression of the tooth preparation using the 3Shape TRIOS^®^ intraoral scanner (3Shape, Copenhagen, Denmark). Subsequently, the students proceeded with the CAD step, designing their own onlay with the assistance of a tutorial video created by the course faculty. Faculty members within the laboratory provided support during the CAD process using the 3Shape TRIOS^®^ Design Studio software (v. 2022.1, 3Shape, Copenhagen, Denmark). The designed onlay was then exported in the Standard Tessellation Language (STL) format, allowing its import into the AnyCubic Workshop (v. 2.1.29, Anycubic, Shenzhen, China) software for 3D printing. In the 3D-printing software, students were able to rotate the model, add support beams, and include an identifying key tag with their name before slicing the model for printing.

**Figure 1:**
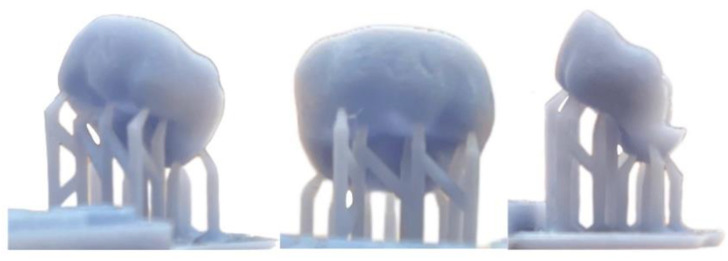
A 3D-printed block immediately after exercise.

The dental students used Photon M2 3D printers (AnyCubic, Shenzhen, China) to successfully perform a CAM exercise by printing unique dental restorations that fit their prepared teeth. The simulation concluded with students seating and adjusting the 3D-printed provisional prosthesis (Figure 1), which was cemented using provisional cement (TempBond NE [non-eugenol], KaVo Kerr, Brea, CA, USA). It is important to note that the hands-on exercise was supplemented with a series of CAD/CAM lectures to provide students with theoretical knowledge alongside the practical application. During the 3D-printing exercise, an additional lecture was conducted in collaboration with the College of Engineering, offering a multidisciplinary teaching approach. A practice management lecture was also delivered to educate students about the clinical considerations when implementing digital dentistry in practice. Following these lectures, an active learning exercise was conducted, where students formed groups to discuss fictional office scenarios and make decisions regarding the acquisition of CAD/CAM technology based on each practice’s profile.

### 2.5. Survey (Questionnaire)

Five parameters guided the development of this survey: (1) knowledge, (2) skill, (3) interest, (4) value of technology, and (5) practice aspiration. The first category included details about the current involvement in the dental field and revealed students’ prior and subsequent knowledge of digital dentistry. The second category reflected the students’ perceptions of digital dentistry. The third category provided insight into students’ attitudes toward digital dentistry. The fourth category verified the relationship between technology and dentistry. The fifth category incorporated a question about the reason(s) for choosing dentistry as a future career, showing their vision as future dentists.

Considering the extent of this exercise and the importance of exposure to CAD/CAM dentistry in a predoctoral academic environment, questions were raised about how this exercise would impact students’ perception of digital dentistry’s value and their aspirations for their professional careers. The survey was anonymous, and the gathered results were organized into a spreadsheet for statistical analysis (Excel v. 16.70, Microsoft Office, Redmond, WA, USA). 

Seven questions were developed, ranging between scores of 1 (lowest) and 10 (highest) or 1 (I strongly disagree) and 10 (I strongly agree), aiming to assess the following areas of interest: (i) change in knowledge, (ii) skill, (iii) interest, (iv) the importance given to technology availability in an office, (v) value of technology to patients, (vi) the importance of having the technology, and (vii) the expected relative frequency of clinics having the technology (Table 2).

### 2.6. Statistical Analysis

The Kolmogorov–Smirnov test evaluated the normality of the data. Sequentially, the Kruskal–Wallis test was used to assess whether control D1, control D3, control D4, and post-D2 were similar. Since D2 may not have been comparable with the controls D1, D3, and D4 in terms of skill and knowledge, those two categories were not tested. However, pre-D2 was analyzed separately since it is not independent of post-D2. Moreover, the Kruskal–Wallis test was applied to compare the difference between post-D2, control D1, control D3, and control D4. To compare the second-year dental students before the exercise (pre-D2) to all the controls combined (negative control [D1] and positive controls [D3 and D4]), Wilcoxon’s test with Bonferroni correction for multiple comparisons was used. Statistical analysis was conducted using the software R (R v3.6.1; R Core Team 2021), and a significance level of 0.05 was assumed.

## 3. Results

### 3.1. Sample

One-hundred eighteen enrolled second-year dental students printed 118 unique dental restorations fitted to their prepared typodont teeth. Then, of the 118 students surveyed, 74 responded (62.71%) to the pre-intervention (pre-D2) and 77 (65.25%) to the post-intervention (post-D2) questionnaire. A total of 120 first-year dental students (D1) were surveyed as negative controls, and 63 responded and participated in this survey (52.5%). Another 240 students were invited (third- and fourth years) as the positive control (120 students from each year), and 43 of D3 (35.83%) and 39 of D4 responded (32.5%). The class composition is shown in Table 3.

### 3.2. Knowledge and Skill

**Figure 2:**
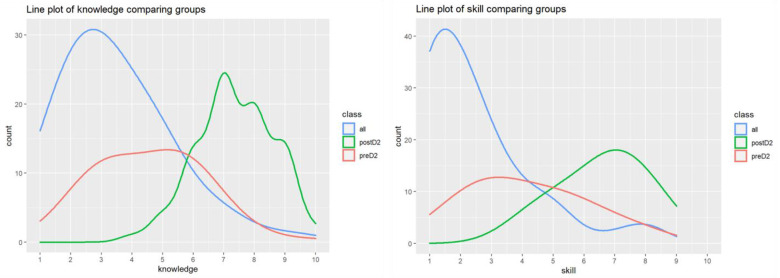
The empirical density of the self-reported knowledge (**left**) and skill (**right**) among all enrolled students.

Analyzing “knowledge” and “skill” (Figure 2), it was noted there was a significant difference between pre-D2 and all the controls (*p* < 0.001 for both). This means that pre-D2 is not comparable with the other controls except for knowledge and skill. Thus, it did not seem appropriate to compare post-D2 with the other controls for the knowledge and skill questions since the difference could not be linked to the intervention.

**Figure 3:**
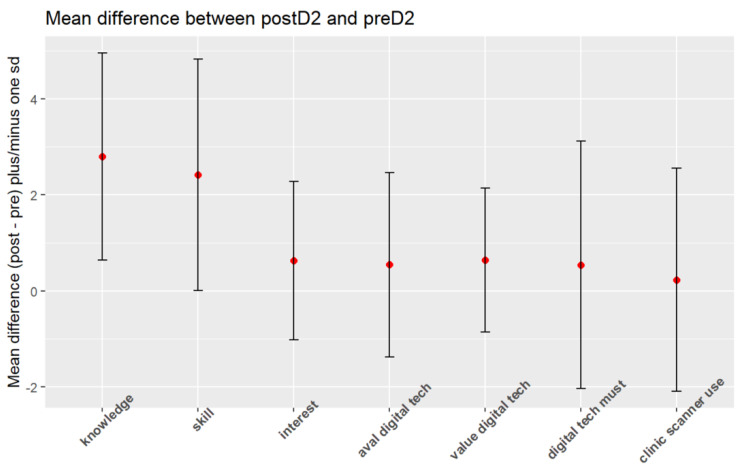
The mean difference for the areas of interest studied, comparing pre-D2 and post-D2.

Multiple comparisons were made to compare the measures between pre-D2 and post-D2 (same students). It was observed there was a significant difference between pre-D2 and post-D2 in “knowledge” (*p* < 0.001) and “skill” (*p* < 0.001) (Figure 3 and Supplementary Figure S1). In addition, multiple comparisons were made for post-D2 and all the control groups (control D1, control D3, and control D4). There was a significant difference between post-D2 and all the controls in “knowledge” (*p* < 0.001) and “skill” (*p* < 0.001). A correction for multiple comparisons was also used to compare post-D2 with control D3 and control D4 combined. A significant difference between post-D2 with control D3 and control D4 combined was found for “knowledge” (*p* < 0.001) and “skill” (*p* < 0.0001).

### 3.3. Interest

The evaluated interest question had a statistically significant result (*p* < 0.05), demonstrating that the pre-D2 students were comparable with all the controls in terms of their interest in digital dentistry. Also, there was a significant difference between post-D2, control D1, control D3, and control D4 for the subject “interest” (*p* = 0.02). For the outcome “interest”, there was a significant difference between post-D2/control D1 (*p* = 0.0362). Multiple comparisons were made to compare the measures between pre-D2 and post-D2 (same students). It was observed there was a significant difference between pre-D2 and post-D2 in terms of “interest” (*p* = 0.0102) (Figure 3 and Supplementary Figure S1). In addition, multiple comparisons were made for post-D2 and all the control groups (control D1, control D3, and control D4). There was a significant difference between post-D2 and all the controls in terms of “interest” (*p* = 0.0127).

**Figure 4:**
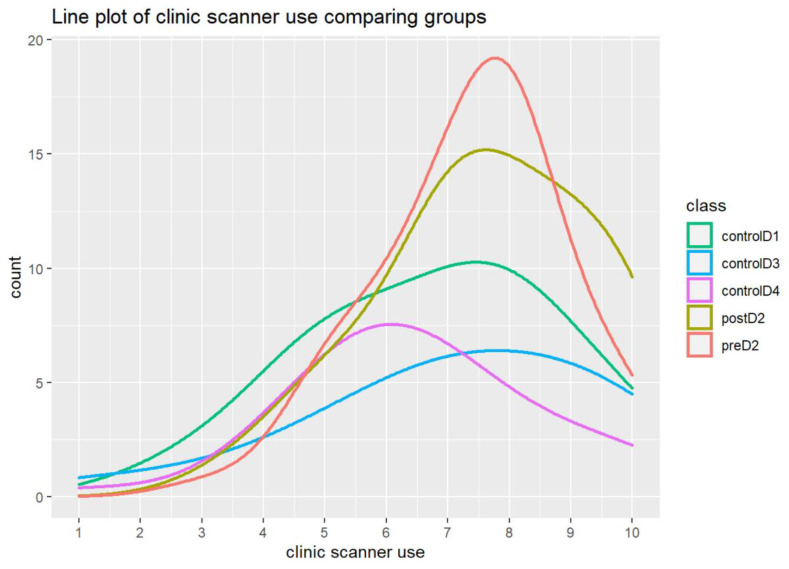
The empirical density of students’ consideration of the “importance of having the technology” for pre-D2/post-D2 and all control groups.

Multiple comparisons were made to compare post-D2 with control D3 and control D4 combined. A significant difference between post-D2 and control D3 and control D4 combined was found in terms of “interest” (*p* = 0.0065). No significant difference was noted between control D1 and control D3 and control D4 combined for all the questions (Figure 4).

### 3.4. Practice Aspiration

The practice aspiration question evaluated had significant results (*p* < 0.05), demonstrating that the pre-D2 students were comparable with all the controls in terms of their preference on the availability of dental technology in their practice. Also, there was a significant difference between post-D2, control D1, control D3, and control D4 for the subject “importance given to technology availability in an office” (*p* < 0.001) and “the relative frequency of clinics having the technology” (*p* = 0.01).

For the outcome “importance given to technology availability in an office”, there was a significant difference between post-D2/control D1 (*p* = 0.00284) and post-D2/control D4 (*p* = 0.0399). No significant difference was found between any of the groups for “the importance of having the technology” (*p* < 0.05). Otherwise, “the relative frequency of clinics having the technology” showed a significant difference between post-D2/control D4 (*p* = 0.01213) (Table 4).

Multiple comparisons were made to compare the measures between pre-D2 and post-D2 (same students). It was observed there was a significant difference between pre-D2 and post-D2 for “importance given to technology availability in an office” (*p* = 0.0024) (Figure 3 and Supplementary Figure S1). In addition, multiple comparisons were made for post-D2 and all the control groups (control D1, control D3, and control D4). There was a significant difference between post-D2 and all the controls for “importance given to technology availability in an office” (*p* < 0.002). 

Multiple comparisons were also used to compare post-D2 with control D3 and control D4 combined. A significant difference between post-D2 with control D3 and control D4 combined was found for “importance given to technology availability in an office” (*p* = 0.0043) and “the relative frequency of clinics having the technology” (*p* = 0.0203). No significant difference was noted between control D1, control D3, and control D4 combined for all the questions (Figure 4).

### 3.5. Value of Technology

All the value parameters evaluated had statistically significant results (*p* < 0.05), demonstrating that the pre-D2 students were comparable with all the controls in this regard. There was no significant difference between any of the groups for “value of technology to patients” and “the importance of having the technology” (*p* < 0.05). Multiple comparisons were made to compare the measures between pre-D2 and post-D2 (same students). It was observed there was no significant difference between pre-D2 and post-D2 for “value of technology to patients” and “the importance of having the technology” (Figure 3 and Supplementary Figure S1). In addition, multiple comparisons were made for post-D2 and all the control groups (control D1, control D3, and control D4). There was no significant difference between post-D2 and all the controls for “value of technology to patients” and “the importance of having the technology”.

Multiple comparisons were also used to compare post-D2 with control D3 and control D4 combined. The results for “value of technology to patients” and “the importance of having the technology” were not significant, and neither were the results for control D1 versus control D3 and control D4 combined for all the questions noted (Figure 4).

## 4. Discussion

The aim of this study was to assess dental students’ perception of digital technologies. The research strategy was to understand whether students’ exposure in a pre-clinical simulation environment to the simulated exercise and scanning, designing, and manufacturing of a computer-aided provisional fixed dental restoration would have a greater impact on the dental students’ future aspiration to incorporate dental technologies into their clinical practice (positive hypothesis). The results highlighted significant changes in students’ knowledge, skill, interest, and technology (importance given to technology availability in an office, value to patients, importance of technology, and the relative frequency of clinics having the technology).

It is relevant to highlight that there were many changes in teaching after the experience of COVID-19 [31]. This fact had tough consequences that will take time to repair. One is the lack of direct interaction with more online classes, causing less contact with patients and reduced work in groups [32,33]. Therefore, the clinical curriculum has undergone modifications appropriate for novice dentists (or the clinic curriculum has undergone appropriate modifications for novice dentists). It is based on new technologies and high-quality evidence of their efficacy. CAD/CAM is one of the most impactful technological advances in dentistry, and its implementation in clinical activities has occurred slowly, mainly when the subject is inserted into the dental curriculum [34]. Since the 2010s, studies have been developed to report on this introduction’s impact on academic behavior. Dehghan et al. (2012) [35] considered the introduction of CEREC (an acronym for “ceramic reconstruction”), which is a CAD/CAM system, at the University of Tennessee College of Dentistry, which was the first university in the US to embrace this technology and integrate it into the fourth-year curriculum. The authors concluded that this technology was an educational tool for dental students, providing cost-effective improvements and exceptional patient service. Browning et al. [36] evaluated undergraduate dental students over one year who provided 125 all-ceramic crowns to patients; they designed, milled, sintered, and stained the CAD/CAM restorations and concluded a significant reduction in lab costs. Also, the authors reported the faculty’s appreciation of the marginal fit and esthetic obtained. The same group published an article in the following year [37] on the same subject to present the incorporation of a CAD/CAM system into the predoctoral curriculum at the Indiana University School of Dentistry. These articles aimed to present data regarding students’ opinions after one year of the implementation. A total of 88 out of 105 D1 students (84% response rate) participated and completed the form. The overall learning was considered good or excellent by 80% of the students, and 43% judged themselves prepared to fabricate a crown independently.

In comparison, the present study’s authors expanded the questionnaire intervention to all students, focusing on the intervention group of second-year students. A total of 118 second-year dental students scanned, designed, and 3D-printed 118 unique dental restorations. The entire group was surveyed, but 74 responded (62.71%) in the pre-intervention (pre-D2) questionnaire and 77 (65.25%) in the post-intervention (post-D2) questionnaire. Positive results were found between pre-D2 and post-D2/all the control groups (D1, D3, and D4) for “knowledge” (*p* < 0.001) and “skill” (*p* < 0.001). Significant differences were also found among post-D2 and the controls D1, D3, and D4 for “technology availability in an office” (*p* < 0.001) and “relative frequency of clinics having the technology” (*p* = 0.01). 

Another study [2], published in 2017, presented the implementation of a CAD/CAM system in the University of Illinois at Chicago College of Dentistry’s predoctoral implant program. The preliminary data showed an increased proportion of implant restorations made digitally compared to traditionally. In 2018, Schweyen et al. [38] evaluated technology implementation in the prosthetic education curriculum at a German dental school. A total of 94% of all students participated in the CAD/CAM curriculum, indicating considerable interest, a number superior to the findings in our study. The restorations fabricated by the students had good clinical performance. The authors concluded that there is a tendency for the use of CAD/CAM systems by the students who prepared digitally more teeth than other students without knowledge of CAD/CAM technology.

In the current study, changes were perceptible in the intervention group (second-year dental students) relative to the negative control (first year) and the positive controls (third and fourth years). Similar to a previous study [1], implementing digital technology was associated with positive student perceptions and attitudes toward future clinical applications. The same study [1] showed that over 90% of students were comfortable with using and willing to use intraoral scanners in their practice. This intervention improved the second-year students’ skills and knowledge in digital dentistry. The skills and knowledge of the control groups were lower than those of the intervention group. In the negative control (first-year students), this may be explained by the fact that those students have not been exposed to or taught digital dentistry yet. For positive controls in the third and fourth years, this may be due to the limited nature of their digital dentistry exposure one or two years ago. Appropriate and timely digital integration into predoctoral education is essential in optimizing the learning environment [2]. The negative control (first-year students) and intervention group (second years) displayed a higher valuation of digital technology in the clinical setting compared to the positive controls (third and fourth years), which may be explained by the lack of digital dentistry in the clinical setting at the time of the survey. This may reflect clinical practice teaching.

The second-year students indicated an increased interest in joining a clinic utilizing dental technology following the intervention exercise. This suggests a change in professional career practice aspiration thanks to this comprehensive exercise. Similar responses were noted in the first-year students and, to a lesser extent, in the third- and fourth-year students. The reduced interest of the positive control group may once again reflect the scarcity of digital technology in the student clinic. All groups expressed high interest in digital dentistry. In the intervention group, this interest was increased. These results are similar to dental students’ positive and enthusiastic attitude towards digital dentistry technology [39].

Towers et al. [40] similarly studied students’ perception of virtual reality (VR) and 3D-printing combinations for operative teaching. Their results complemented this study, highlighting the value of technology to students and innovative teaching methods that are translatable into clinical settings. The study also highlighted the importance of educator support, which was not assessed in this survey. The impact of this teaching on the clinical procedure and the patient is notable in this study, mainly in providing a valuable contribution to increasing students’ confidence and preparedness.

Finally, one study published in 2023 [41] aimed to evaluate predoctoral dental students’ CAD/CAM-related education, knowledge, attitudes, and professional behavior; moreover, the relationships between years in dental school and other variables were contrasted. A total of 358 dental students from 17 of the 68 US dental schools (25%) participated in a web-based anonymous survey. Similar to the present study, the questions asking about particular subjects and the percentages obtained were simulated exercises (86.9%), video demonstrations (81.8%), demonstrations during a lecture (76.4%) or to smaller groups of students (69.2%), hands-on (65.6%), and individual instruction (50.4%). There was a significant improvement in the knowledge and attitude to using CAD/CAM technology (*p* < 0.001 and *p* < 0.05, respectively); otherwise, student satisfaction was non-significant. The authors concluded that most students in US dental schools considered CAD/CAM to be the future of dentistry and that it made them better dentists.

### 4.1. Study Limitation

There are limitations to consider. Limited and concentrated students’ opinions were obtained, which might not translate into students’ skills. Moreover, students’ perceptions of concepts, such as skills and knowledge, can inflate responses to questions. Only one institution was involved, although similar experiences were conducted in other institutions during analog-to-digital transfer. The focus could be amplified to all years, not only second year, to observe the maintenance of learning and the knowledge reached in the superior years (third and fourth). Moreover, to ensure the anonymity of the respondents, no identification was recorded from any of the applied questionnaires. Consequently, we could not pair repeated measures from the second-year participants before and after the intervention, needing to assume independence instead.

### 4.2. Recommendations for Future Studies

Further research (prospective cohort study) is suggested to follow up a greater number of students in the long term, indicating a multicentric design. Moreover, by extending the subject of this study, which was to assess dental students’ perceptions of digital technologies and scanning, designing, and manufacturing a computer-aided provisional fixed dental restoration, more studies can approach new trends in dental materials and other technologies, such as making zirconia crowns or testing other resin materials to evaluate their mechanical properties and artificial aging’s influence, their tensile modulus, the influence of polishing on their surface texture, the effect of thermocycling on the resins, and their fractal dimensions, in the hope of updating readers and professionals [42].

## 5. Conclusions

Within the limitations of this study, it was possible to conclude that (i) around 60% of the second-year students (experimental group) responded to the questionnaire, with statistically significant results for the subject “interest”; (ii) a significant improvement was observed for “knowledge” and “skill” when comparing the pre-D2 and post-D2/all control groups (control D1, control D3, and control D4); (iii) a significant impact was found on the answers on the presence of technology in practice and the educational academic environment.

## Figures and Tables

**Table 1:** Eligibility criteria summarized.

Inclusion Criteria	Exclusion Criteria
- Students at the OSU College of Dentistry - Completed the activity - Filled out the surveys - Students: 1st year (negative control) 2nd year (test group) 3rd and 4th years (positive control)	- Students in other medical areas or different fields - Researcher or post-doc students at the OSU College of Dentistry - Rejected filling out survey - Did not complete the activity

**Table 2:** The questionnaire applied to the students (1 lowest, 10 highest or 1 I strongly disagree, 10 I strongly agree).

I would rate my knowledge level in digital dentistry as I would rate my skill level in digital dentistry as I would rate my interest level in digital dentistry as The availability of digital technology in a clinic will highly impact my decision to practice there or not Patients will value the use of dental technology Digital technology is “a must” in any dental clinic Most, if not all, clinics are now using scanners

**Table 3:** Class compositions.

Class	Class Size	Age Range/Average	Gender			Underrepresented Minority
			Male	Female	Other	
2023	120	21–34/24	65	55	1	8
2024	120	19–50/23	54	66	0	13
2025	118	20–38/22	55	63	0	14
2026	120	19–41/22	55	65	0	5

**Table 4:** Summary statistics.

	Post D2	Pre D2	Control D1	Control D3	Control D4	D3/D4	Control
*n*	77	74	63	43	39	82	145
knowledge (mean (SD))	7.38 (1.26)	4.58 (1.75)	3.68 (2.18)	3.51 (1.53)	3.15 (1.44)	3.34 (1.49)	3.49 (1.82)
skill (mean (SD))	6.57 (1.46)	4.15 (1.91)	2.60 (2.11)	2.70 (1.49)	2.13 (1.47)	2.43 (1.50)	2.50 (1.78)
interest (mean (SD))	9.45 (0.90)	8.82 (1.39)	8.79 (1.56)	8.84 (1.40)	9.13 (0.95)	8.98 (1.21)	8.90 (1.37)
aval_digital_tech (mean (SD))	8.90 (1.30)	8.35 (1.41)	7.78 (2.02)	8.19 (1.79)	7.79 (2.14)	8.00 (1.96)	7.90 (1.98)
value_digital_tech (mean (SD))	9.49 (0.87)	8.85 (1.22)	9.05 (1.30)	9.33 (1.02)	9.10 (1.19)	9.22 (1.10)	9.14 (1.19)
digital_tech_must (mean (SD))	8.30 (1.86)	7.76 (1.78)	7.78 (2.18)	8.02 (2.06)	7.82 (2.02)	7.93 (2.04)	7.86 (2.09)
clinic_scanner_use (mean (SD))	7.60 (1.76)	7.36 (1.51)	6.79 (2.01)	7.12 (2.38)	6.49 (1.92)	6.82 (2.18)	6.81 (2.10)

## Data Availability

All data were inserted in the article.

## Supplementary Materials

The following supporting information can be downloaded at https://www.mdpi.com/article/10.3390/dj12030075/s1. Figure S1: Box plot for the areas of interest studied, comparing pre-D2 and post-D2.
