# Supplementary material for: The Impact of Technology Teaching in the Dental Predoctoral Curriculum on Students’ Perception of Digital Dentistry
Source: Dent J (Basel). 2024 Mar 13;12(3):75. doi: 10.3390/dj12030075 (PMC10969759; doi:10.3390/dj12030075)
Supplement: Supplementary file 1 [file dentistry-12-00075-s001.zip › dentistry-2836247-supplementary.pdf]

Supplementary Figure

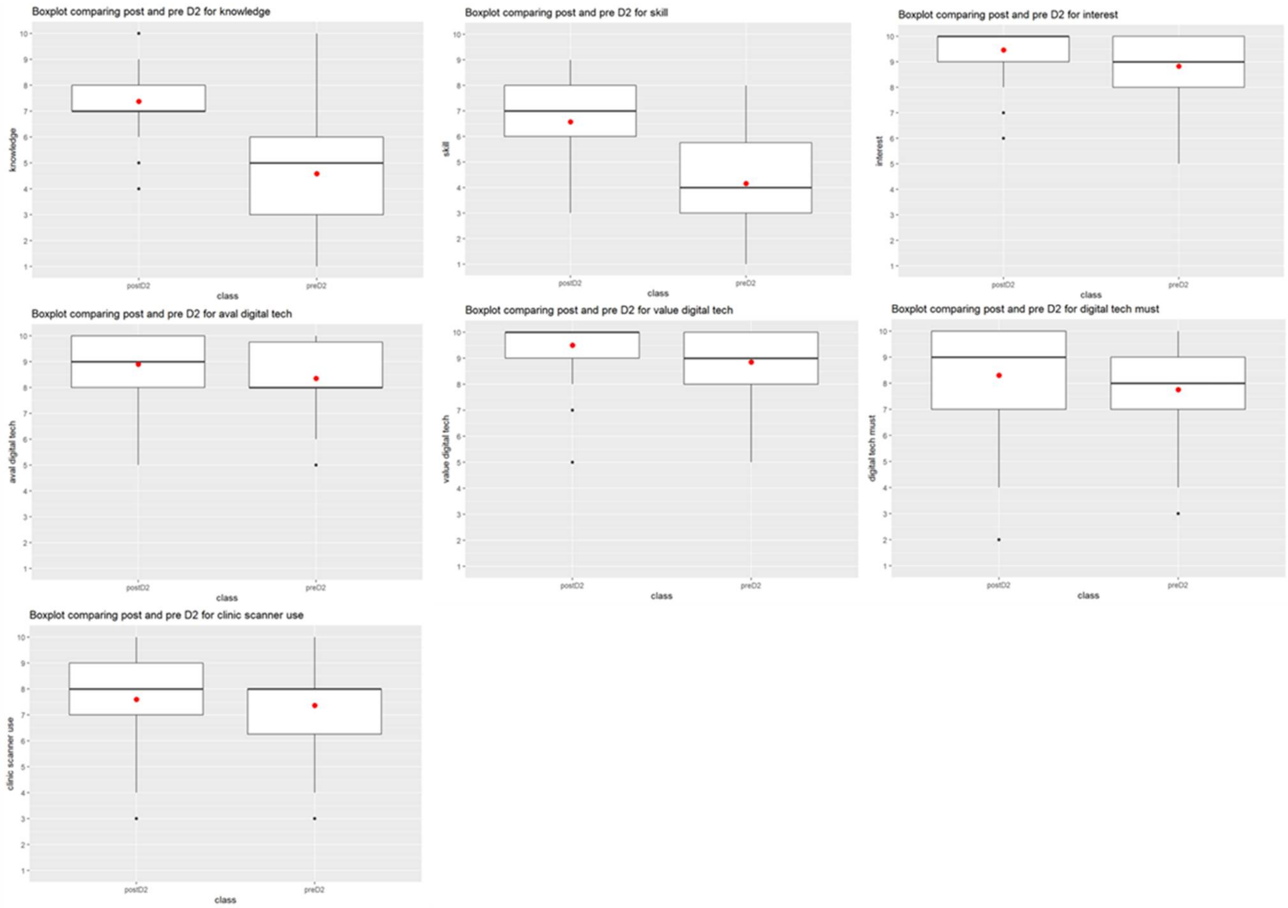

Suppl. Figure S1. Box plot for the areas of interest studied, comparing pre-D2 and post-D2.
